# Bioreactor Optimisation Through Coupled Fluid–Structure Interaction and Data-Driven Analysis: A Biomimetic Framework for Predicting Early Cell Attachment

**DOI:** 10.3390/biomimetics11100742

**Published:** 2026-10-15

**Authors:** Yuyang Wei, Weijie Fei, Jiarong Wang, Luzheng Bi

**Affiliations:** School of Mechanical Engineering, Beijing Institute of Technology, Beijing 100081, China; yuyang.wei@bit.edu.cn (Y.W.); bitfwj@bit.edu.cn (W.F.)

**Keywords:** biomimetic bioreactor, fluid–structure interaction, machine learning, multilayer perceptron, cell attachment, wall shear stress, scaffold stiffness, tissue engineering, perfusion culture, humanoid robotic loading

## Abstract

Early cell attachment sets the baseline for proliferation, matrix deposition and construct maturation in tissue engineering, yet conventional bioreactors deliver simplified, quasi-static stimuli that poorly represent the multi-axial mechanical environment of native tissue. Here we reported a biomimetic computational–experimental framework for a humanoid-robot-driven perfusion bioreactor. The framework coupled a high-fidelity fluid–structure interaction (FSI) model of the culture chamber with a machine-learning model in order to describe the initial 3 h attachment phase, which preceded the application of robotic multi-axial loading. All experimental data reported here were therefore acquired during the static and perfused seeding phase; robotic loading was applied subsequently and was not part of the present dataset. Local mechanical and fluidic fields—wall shear stress, flow velocity, pressure, von Mises stress and scaffold strain—were mapped across ten scaffold subregions and paired with spatially resolved cell numbers obtained by PicoGreen double-stranded DNA quantification. Six regression algorithms were compared under identical conditions; a multilayer perceptron (MLP) gave the lowest mean absolute error (13.2%). Permutation importance ranked flow velocity, wall shear stress, von Mises stress and pressure as the dominant correlates of early adhesion. Parameter sweeps of perfusion rate and scaffold stiffness were model predictions rather than measured optima, and identified 1.4–1.6 mL/min and a moderately compliant scaffold as candidate operating points for experimental testing. The framework provides a transferable route to rational bioreactor and scaffold tuning with reduced empirical trial and error.

## 1. Introduction

Tissue engineering has emerged as a transformative field in regenerative medicine, aiming to repair or replace damaged tissues by cultivating living cellular constructs in controlled laboratory environments [1]. Central to the success of these engineered tissues is the ability to recreate the precise mechanical, biochemical and physiological cues of native tissues [2]. However, achieving such fidelity remains highly challenging because of the inherent complexity and dynamic nature of biological systems.

One of the most powerful tools driving progress in this area is the bioreactor—a specialised device designed to support and regulate tissue growth by delivering controlled mechanical forces, nutrient flow and environmental conditions [3]. Through carefully tuned perfusion, mechanical stimulation and nutrient delivery, bioreactors can mimic aspects of in vivo physiology, enhancing cell attachment, proliferation, differentiation and overall tissue maturation [4]. Yet conventional bioreactor designs often fall short in replicating the dynamic and heterogeneous mechanical environments found in the body. Many still rely on simplified or static stimuli, such as uniform shear stress or unidirectional strain, which inadequately capture the multi-axial and time-varying biomechanical cues that cells naturally experience [5].

To address these limitations, computational modelling—particularly fluid–structure interaction (FSI) simulation—has become a powerful approach for optimising bioreactor systems [6]. FSI integrates computational fluid dynamics (CFD) with structural mechanics to provide detailed insight into how fluid flow interacts with scaffold deformation, enabling researchers to explore how coupled mechanical and fluidic parameters influence cell behaviour [7]. However, earlier FSI implementations often adopted oversimplified assumptions—uniform strain distributions, linear flow models or static load conditions—that fail to capture the highly dynamic and spatially heterogeneous nature of real tissue environments.

Previous work has shown that FSI-based design can improve nutrient delivery and scaffold architecture [8]. Nevertheless, such studies frequently omit important biomechanical complexities, such as those arising from physiologically relevant joint kinematics or cyclic mechanical loading [9]. Furthermore, most computational frameworks do not fully integrate artificial intelligence (AI) or machine learning (ML), despite their ability to model complex nonlinear relationships between mechanical stimuli and biological responses [10].

Taken together, the literature leaves three specific gaps that motivate the present study. First, bioreactors that impose genuinely multi-axial, joint-like kinematics have been described mechanically, but the local fields they generate have not been resolved region by region and linked quantitatively to a measured biological endpoint. Second, where FSI and ML have been combined in bioprocess work, the ML layer has typically been applied to lumped, chamber-averaged quantities, so that it cannot distinguish which local field drives a spatially heterogeneous response. Third, and most importantly for design practice, the seeding phase that determines the initial spatial distribution of cells is rarely treated as an explicit optimisation target, even though every later culture stage inherits that distribution. The present work addresses these three gaps by resolving the local fields region by region, by pairing each region with its own measured cell number, and by restricting the analysis to the seeding window in which the measurement was actually made.

In this context, we presented a biomimetic computational–experimental framework that combined high-fidelity FSI simulation with ML-based predictive modelling, focused on the initial (3 h) cell attachment phase. This early stage is critical because it sets the baseline for subsequent cell proliferation, extracellular matrix deposition and tissue maturation. The bioreactor incorporates a humanoid robotic arm that reproduces physiologically relevant joint motions—such as adduction, abduction and complex rotations—in the culture phase that follows seeding. Because the attachment assay used here was completed before robotic loading began, the mechanical environment analysed in this paper is that of the static and perfused seeding phase; the robotic loading stage is described for completeness and is the subject of ongoing work. We systematically evaluated multiple ML algorithms, including random forest, XGBoost, k-nearest neighbours, support vector regression, linear regression and the multilayer perceptron (MLP), to identify the most accurate model for mapping biomechanical inputs to early-stage biological outcomes.

To ensure real-world relevance, we validated the computational predictions against experimental measurements obtained by fluorescence-based PicoGreen DNA quantification, which enabled precise, spatially resolved analysis of cell distribution across the scaffold. This integration of physiologically realistic mechanics, data-driven modelling and experimental validation creates a predictive framework that can guide the design of bioreactors for regenerative therapies and rehabilitation-oriented tissue culture systems.

## 2. Materials and Methods

In this study, the primary focus was the prediction of the initial cell attachment phase, specifically at the 3 h time point after seeding. This early stage is biologically critical, as it sets the foundation for downstream cellular behaviours such as proliferation, alignment and extracellular matrix deposition. Prior studies have shown that the first few hours following cell seeding are highly sensitive to local mechanical and fluidic cues, which can significantly influence the success of long-term tissue formation. By targeting this window, we aimed to identify the design and operating conditions that support robust early cell adhesion.

**Figure 1:**
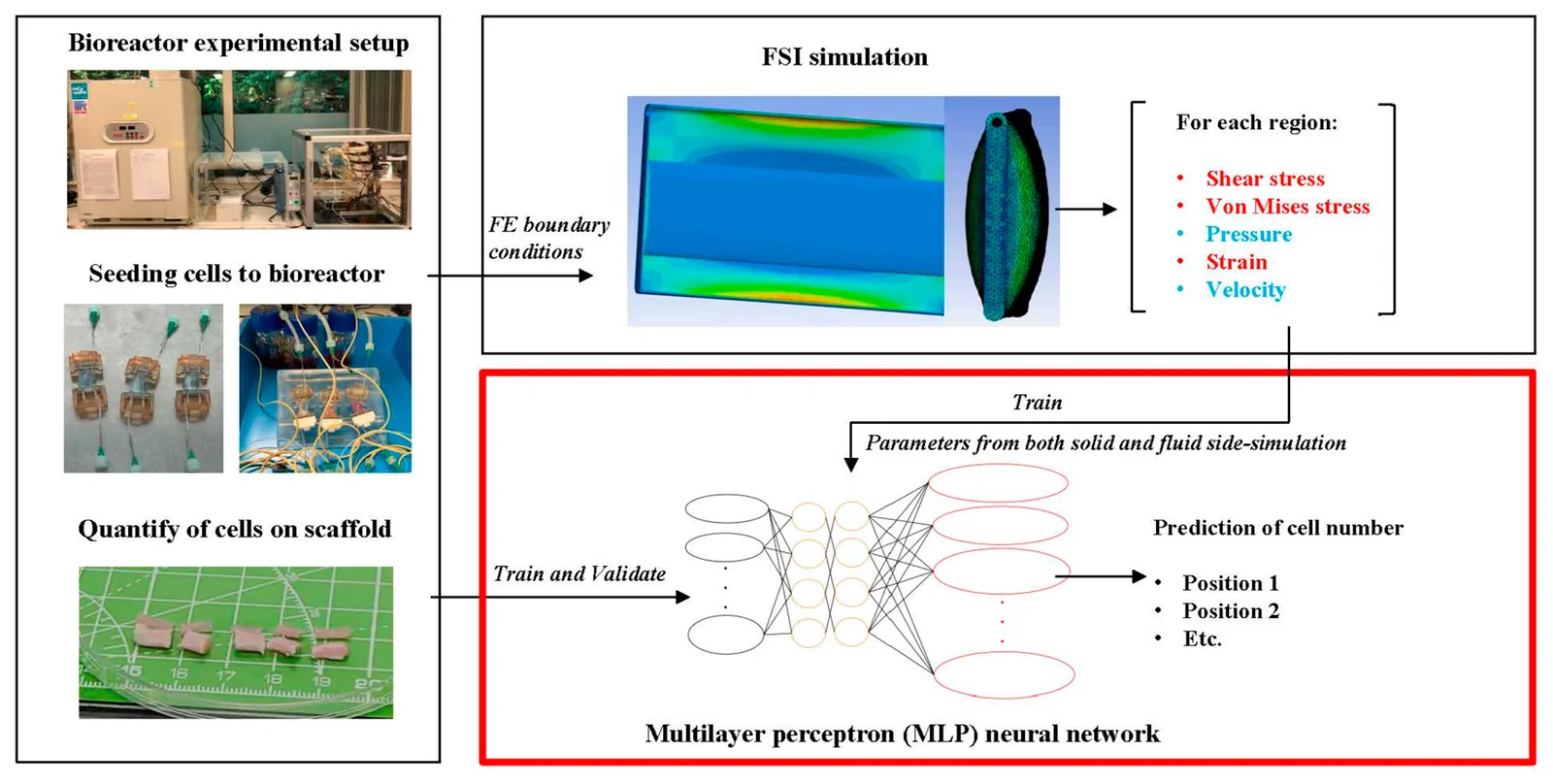
Overview of the integrated computational–experimental framework. Experimental inputs (bioreactor setup, cell seeding and region-wise quantification of cells on the scaffold) define the boundary conditions of the fluid–structure interaction (FSI) simulation. For each of the ten scaffold subregions, wall shear stress (Pa), von Mises stress (Pa), pressure (Pa), equivalent elastic strain (–) and flow velocity (m/s) are extracted and used to train and validate a multilayer perceptron (MLP) neural network that predicts the region-specific cell number. The experimental panels correspond to the static and perfused seeding phase, which is the phase analysed in this paper.

As shown in Figure 1, a numerical framework was established that integrated finite element (FE) fluid–structure interaction simulation with machine-learning prediction in order to investigate the relationship between local mechanical environments and cell attachment.

### 2.1. Bioreactor Experimental Setup and Experimental Timeline

The bioreactor system integrates a humanoid robotic arm with a flexible bioreactor chamber, designed to reproduce physiological tissue movements. This configuration aims to enhance the maturation and functionality of tissue-engineered constructs by applying biomechanically relevant stimuli that mirror human joint dynamics. The bioreactor is equipped with a control system that allows precise adjustment of mechanical stimuli, including tension, compression and torsion, which are essential for cellular mechanotransduction and tissue development.

**Figure 2:**
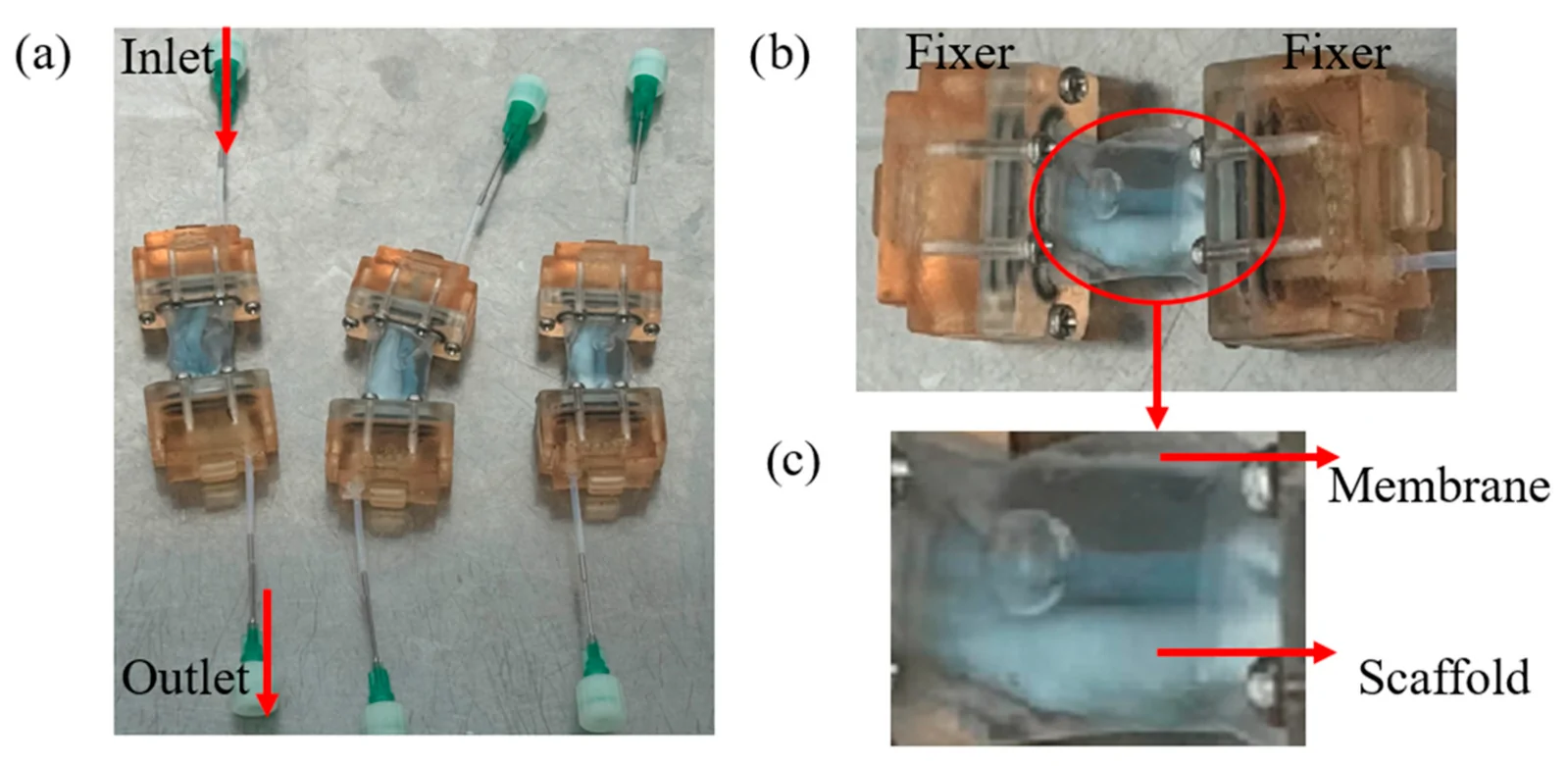
Custom-designed perfusion bioreactor used in this study. The chamber was developed for tendon-like constructs; in the present seeding study it was seeded with human foreskin fibroblasts (HFF-1), which were used as a well-characterised fibroblast model for the attachment assay rather than as tendon-derived cells. (**a**) The system includes an inlet and an outlet for fluid perfusion. (**b**) Fixtures securing the membrane structure and the internal chamber housing the scaffold. (**c**) The scaffold positioned within a flexible membrane that mimics physiological boundary conditions. The design allows precise control of flow rate and mechanical cues during perfusion.

The experimental sequence was as follows. (i) Seeding and attachment phase, 0–3 h: the chambers were held in an incubator and connected to a peristaltic pump that circulated culture medium from a reservoir through an oxygenator and back to the chamber (Figure 2); the robotic arm was not engaged during this phase. (ii) Attachment assay at t = 3 h: the constructs were removed and processed for region-wise dsDNA quantification, so that the 3 h endpoint reported here terminates the seeding phase. (iii) Maturation phase, t > 3 h: separate constructs were transferred to the humanoid robotic arm for cyclic multi-axial loading. All attachment data, all FSI fields and all machine-learning results presented in this paper belong to phase (i), and the robotic loading of phase (iii) could not have influenced them. The maturation phase is described here only to document the purpose of the device and will be reported separately.

For completeness, the loading protocol of phase (iii) consisted of programmed adduction–abduction cycles with an angular amplitude of ±15°, a frequency of 0.5 Hz and a daily duration of 1 h per day for 7 days.

**Figure 3:**
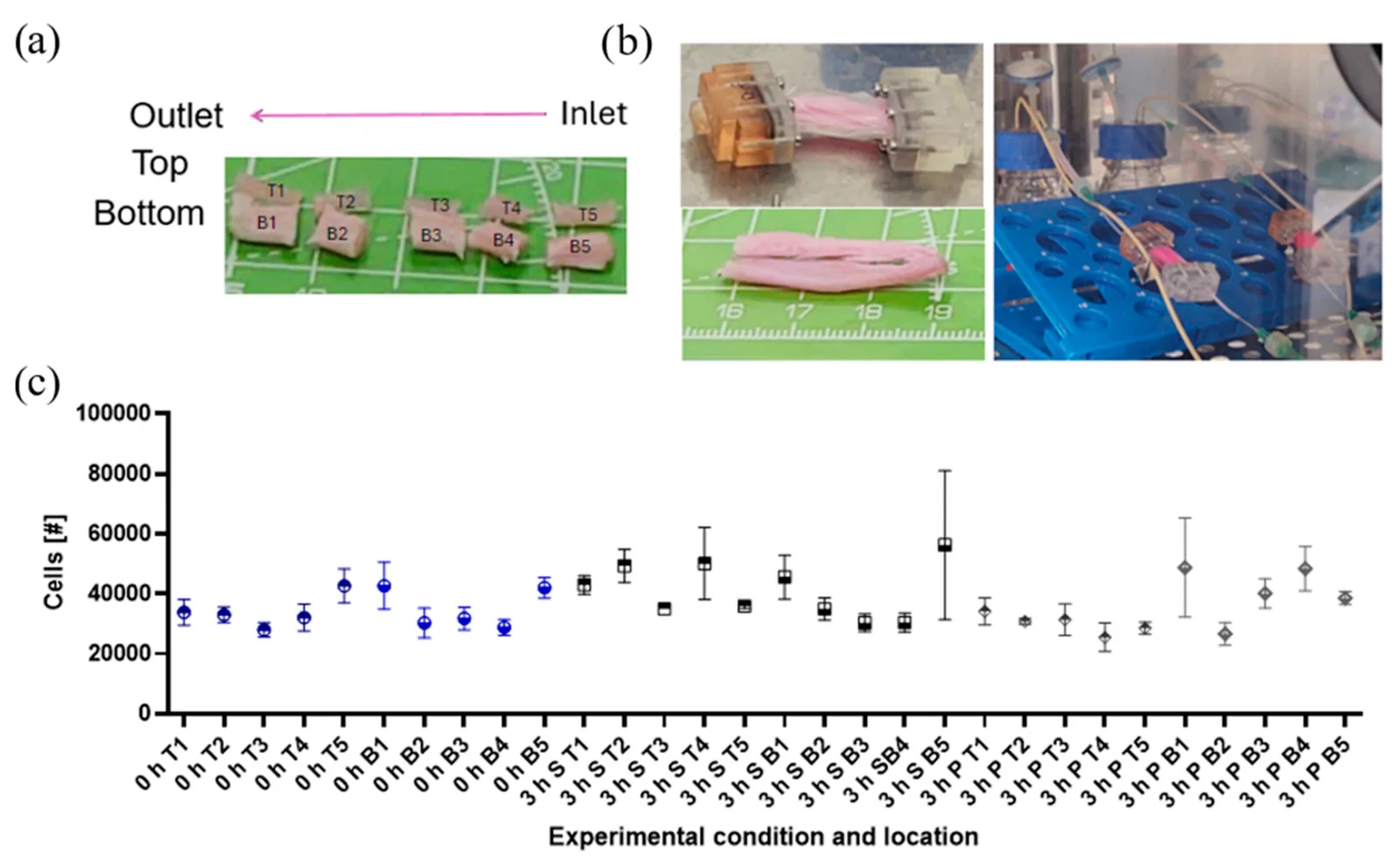
Quantification of cell attachment and distribution on the scaffold using the PicoGreen dsDNA assay. (**a**) Mapping of the top (T1–T5) and bottom (B1–B5) scaffold regions along the flow direction from inlet to outlet. (**b**) Experimental setup for cell seeding and perfusion inside the bioreactor. (**c**) Quantified cell numbers (cells per subregion) at each location and time point (0 h and 3 h) for the three conditions S (static, no perfusion), P (perfusion, 1.5 mL/min) and HP (high perfusion, 3.0 mL/min). Error bars represent the standard deviation across independent constructs (*n* = 3 per condition).

The three conditions compared in Figure 3 are defined as follows: S denotes the static condition, in which the chamber was held without perfusion; P denotes perfusion at the baseline flow rate of 1.5 mL/min; and HP denotes high perfusion at 3.0 mL/min. The S condition at 3 h therefore serves as the non-perfused control against which the perfused spatial patterns are interpreted, and the 0 h data provide the corresponding seeding baseline.

This two-step process leverages the advantages of both static and dynamic culture environments. By first allowing the cells to acclimatise and attach and then introducing biomechanically relevant stimuli, the experimental protocol reproduces the natural developmental sequence of tissue formation.

### 2.2. DNA Labelling and Cell Quantification

Accurate quantification of cellular distribution on the scaffold is essential for validating the FSI and machine-learning predictions. A fluorescence-based PicoGreen assay was used, which is specific and sensitive to double-stranded DNA (dsDNA) and therefore provides robust quantification of cell number (Figure 3).

Human foreskin fibroblasts (HFF-1 cell line; ATCC, Manassas, VA, USA; Cat. No. SCRC-1041) were cultured and expanded in Dulbecco’s modified Eagle medium (DMEM) supplemented with 10% foetal bovine serum (FBS) and 1% antibiotic–antimycotic solution, at 37 °C in a 5% CO_2_ incubator. Before seeding onto the scaffolds, cells were detached with 0.25% trypsin–EDTA, counted using a haemocytometer and resuspended in fresh culture medium. Each scaffold was seeded with 5.0 × 10^5^ cells per scaffold in 500 µL of medium, applied uniformly over the upper scaffold surface. Following seeding, the construct was maintained in the incubator for 3 h. The scaffolds were then removed from the bioreactor and divided into ten distinct, equally sized subregions using a sterile scalpel, as illustrated in Figure 1. The ten subregions comprised five positions along the flow axis on the upper surface (T1–T5) and the five corresponding positions on the lower surface (B1–B5), so that the upper and lower faces were processed and analysed separately rather than pooled.

Each subregion was immediately transferred into an individual sterile tube for DNA extraction, which was performed with a commercial kit (DNeasy Blood & Tissue Kit, Qiagen, Hilden, Germany) according to the manufacturer’s instructions. Extracted DNA was quantified by staining with PicoGreen reagent: samples were incubated with diluted PicoGreen staining solution for 5 min at room temperature in darkness, and fluorescence intensity was then measured using a microplate fluorometer (BioTek Instruments, Winooski, VT, USA) at excitation and emission wavelengths of 485 nm and 530 nm. Fluorescence intensities were converted to DNA concentrations using a standard curve constructed from the DNA standards supplied with the assay kit. Three independent constructs were analysed per condition (*n* = 3).

Two further controls were applied so that the reported cell numbers reflect attached cells rather than retained free DNA or extraction losses. First, the conversion from dsDNA mass to cell number used a calibration curve built from known HFF-1 cell numbers processed through the identical extraction protocol, giving 5.8 ± 0.4 pg dsDNA per cell; because the calibration cells were carried through the same extraction, the factor already incorporates the extraction efficiency. Second, the recovery after sectioning the scaffold into ten pieces was assessed by comparing the summed dsDNA of the ten subregions with that of intact control scaffolds, which gave a recovery of 91.4 ± 3.2%. Unattached cells were removed by rinsing the construct before sectioning, and the static (S) condition at the same 3 h endpoint provides the sedimentation-only reference needed before any spatial pattern is attributed to flow.

Finally, the DNA concentration obtained from each scaffold subregion was converted into a cell number using the calibration described above. This procedure provided region-specific quantification of the cell distribution on the scaffold, which was used as the ground truth for the numerical predictions.

### 2.3. Numerical Framework: Fluid–Structure Interaction Layer

#### 2.3.1. Three-Dimensional Geometry of the FSI Model

**Figure 4:**
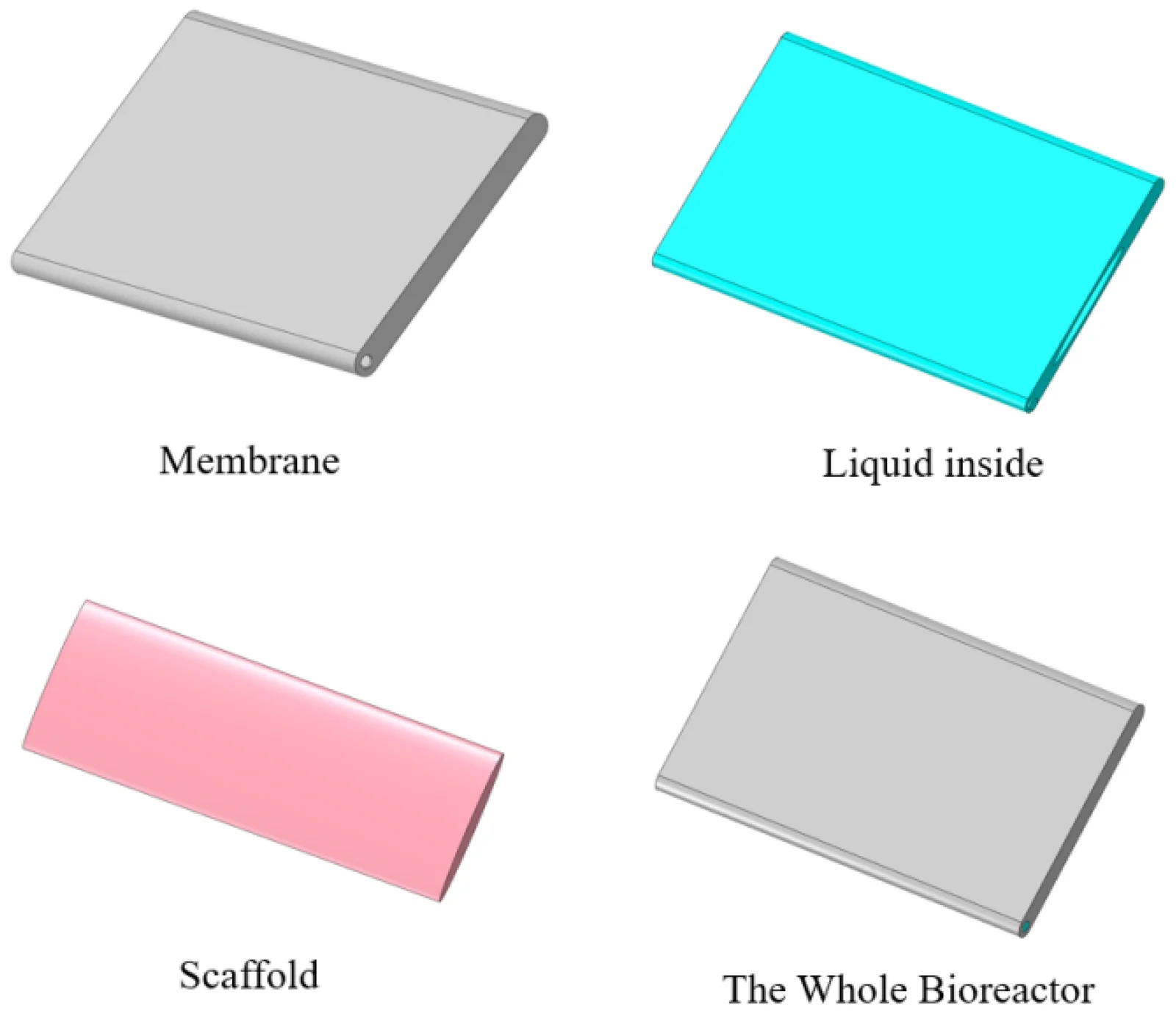
Components of the three-dimensional FSI model: the flexible membrane, the internal fluid domain, the scaffold and the assembled bioreactor. The scaffold is represented as an impermeable deformable continuum (Section 2.3.1).

The three-dimensional geometry of the FSI model (Figure 4) reproduces the key components of the bioreactor, ensuring an accurate representation of the interactions between scaffold, membrane and fluid [11].

**Membrane.** The membrane was modelled as a flat rectangular chamber of width 5 cm and length 10 cm, whose depth is small relative to its in-plane dimensions. It is made of a flexible, biocompatible material and reproduces the mechanical behaviour of the chamber under load.

**Scaffold.** At the core of the bioreactor, the scaffold has a width of 3 cm and a length of 8 cm. The physical scaffold is a decellularised porcine tendon matrix whose porosity permits cell infiltration and nutrient exchange [12].

**Representation of the scaffold in the model.** In the present simulations the scaffold was represented as an impermeable deformable continuum, and the medium was resolved as clear fluid flowing over and around it rather than through it. No Darcy or Brinkman term was therefore included, and no permeability or porosity value entered the model. This is a deliberate simplification, chosen because the quantity paired with the biological measurement is the field at the scaffold surface, where the cells were seeded and where they were quantified. Its consequence is that intra-scaffold perfusion and pore-scale shear gradients are not resolved, so the computed wall shear stress should be read as the surface-level shear experienced by the seeded face and not as the shear within the pore network [13]. Section 4.6 sets out this limitation and the poroelastic extension that would remove it.

**Fluid domain.** The fluid domain surrounding the scaffold, bounded by the membrane, governs the flow field within the chamber. The simulation accounts for the density and viscosity of the culture medium in order to model shear stress and pressure distribution as the fluid interacts with the scaffold [14].

#### 2.3.2. Material Property Definition

In the finite element simulation, the bioreactor membrane was modelled as a linear elastic polyurethane with a Young’s modulus of 6 MPa and a density of 1.21 g/cm^3^.

The decellularised porcine tendon scaffold was represented by an isotropic, nearly incompressible Neo-Hookean hyperelastic model, with an incompressibility parameter D of 0.00683 MPa^−1^. Two shear moduli reported for human ligament grafts were available to us: 58.23 MPa for bone–patellar tendon–bone and 38.74 MPa for semitendinosus grafts. The simulations reported in this paper used the semitendinosus value, μ = 38.74 MPa, as the baseline. Because the material is treated as incompressible, the shear modulus and the Young’s modulus are not independent but are related by E = 3μ, so the baseline corresponds to E = 116.2 MPa. The stiffness levels in Table 3 are scalings of this single baseline parameter and therefore scale μ and E together; the percentages do not refer to a second, independent modulus.

We note explicitly that this constitutive choice is a simplification. Native tendon is a transversely isotropic, fibre-reinforced and strongly hierarchical material whose longitudinal modulus greatly exceeds its transverse and shear moduli, and whose apparent stiffness depends on the measurement modality: quasi-static tensile testing of the collagenous phase yields moduli in the hundreds of MPa, whereas shear-wave elastography of hydrated tissue yields values orders of magnitude lower [15]. The isotropic Neo-Hookean description used here is intended only to reproduce the bulk compliance of the construct at the small strains imposed during perfusion (equivalent elastic strain below 3.6 × 10^−8^), which is the regime that determines the chamber deformation and hence the flow field. It is not intended as a constitutive description of tendon, and it is not appropriate for the large-strain, fibre-aligned loading of the maturation phase. An anisotropic hyperelastic or poroelastic formulation would be required for that regime and is identified in Section 4.6 as the principal modelling priority for future work.

#### 2.3.3. Boundary Conditions, Fluid Properties and Numerical Settings

The boundary conditions were defined to represent the dynamic interaction between the culture medium and the scaffold.

**Figure 5:**
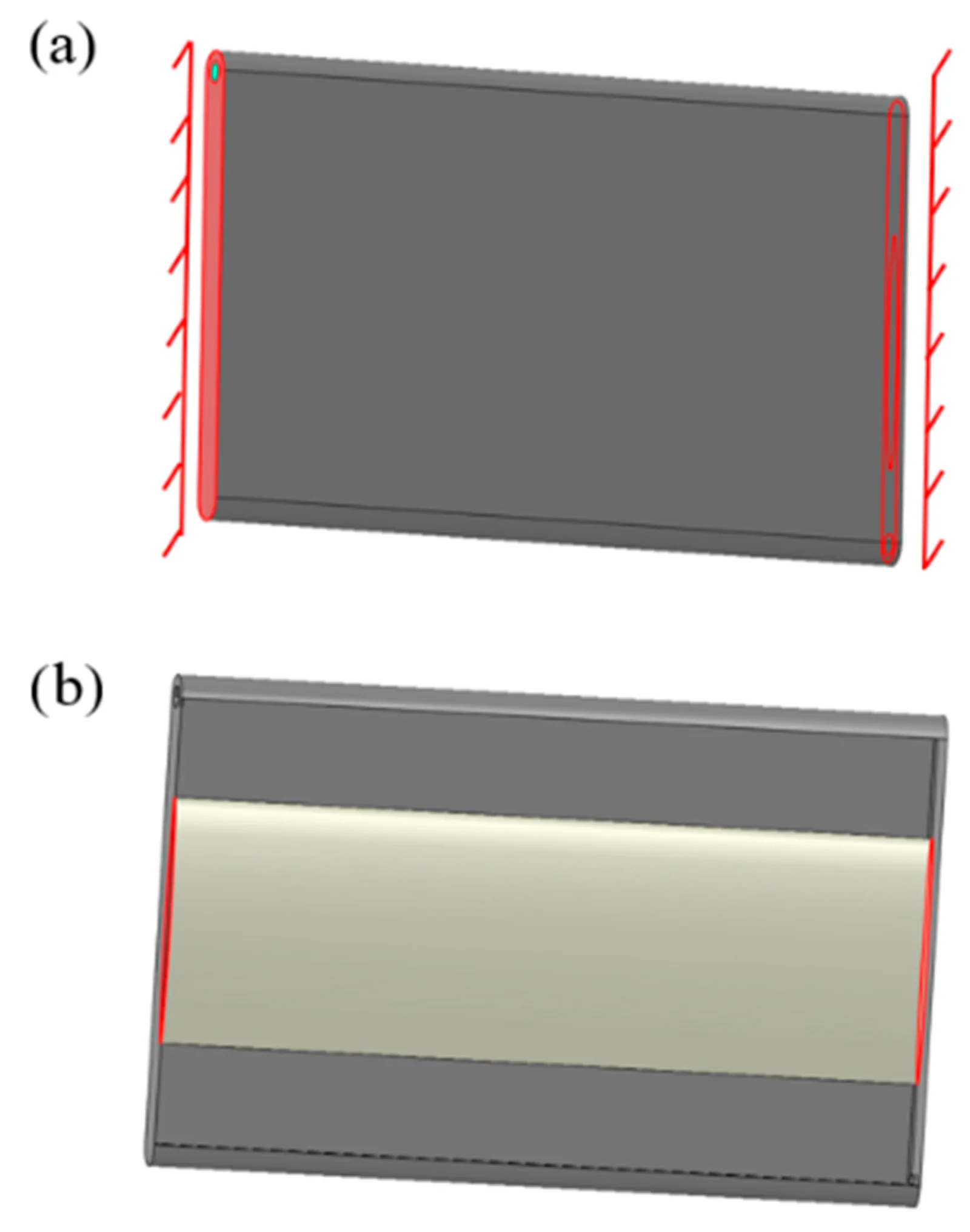
Boundary conditions applied in the FSI model. (**a**) Two opposite sides of the membrane (red) are fully constrained, representing attachment to the bioreactor housing. (**b**) The scaffold is tied to the membrane along two sides, so that the scaffold and membrane deform as a single entity.

As illustrated in Figure 5, two sides of the membrane were fully fixed, preventing translation and rotation; this represents the membrane being securely attached to the bioreactor housing. Two sides of the scaffold were tied to the membrane so that the two components move together as a single entity.

A uniform inlet velocity corresponding to a volumetric flow rate of 1.5 mL/min was specified at one boundary of the fluid domain, using a magnitude-normal-to-boundary specification. A pressure boundary condition of 0 Pa was applied at the outlet. A no-slip wall condition was applied at the solid–fluid interface. The roughness constant of 0.5 used at these walls is the dimensionless constant C*s* of the Fluent sand-grain roughness model and is the solver default; it is not a roughness height. The associated physical roughness height K*s* was set to zero, i.e., the walls were treated as hydraulically smooth, so the roughness model was inactive in these simulations.

The culture medium was modelled as an incompressible Newtonian fluid with a density of 1009 kg/m^3^ and a dynamic viscosity of 7.8 × 10^−4^ Pa·s at 37 °C. The Newtonian assumption is appropriate here even though the medium is supplemented with 10% FBS: the suspended protein fraction of serum-supplemented DMEM is dilute, and measurements of such media report a viscosity close to that of water at 37 °C with no measurable shear-thinning over the shear-rate range relevant to perfusion culture [16]. Any residual rate dependence is small compared with the uncertainty introduced by the geometric and constitutive simplifications discussed above. The cell suspension itself was not modelled as a separate phase.

With these properties, the Reynolds number based on the hydraulic diameter of the chamber and the mean inlet velocity was 1.2, confirming that the flow was laminar throughout the operating range examined; a laminar viscous model was therefore used.

The fluid problem was solved in ANSYS 2024 R1 Fluent and the structural problem in ANSYS Mechanical, coupled through the System Coupling module using a two-way scheme, with data exchanged at the fluid–solid interface and up to 15 coupling iterations per step at a convergence tolerance of 1 × 10^−3^ (RMS) on the transferred force and displacement. The incompressible Navier–Stokes equations were solved with the semi-implicit method for pressure-linked equations (SIMPLE) algorithm [17], and the governing equations [18] were discretised using the finite volume method [19]:

(1) ∇ · *u* = 0,

(2) ∂*u*/∂*t* + (*u* · ∇)*u* = −(1/*ρ*)∇*p* + *ν*∇^2^*u*,

where *u* is the velocity vector field, *p* is the pressure, *ρ* is the fluid density, *ν* is the kinematic viscosity and ∇ is the gradient operator. Equations (1) and (2) are the clear-fluid form; consistent with Section 2.3.1 they contain no porous-resistance term.

Mesh and step independence were established as follows. The fluid and solid domains were discretised with 1.42 × 10^6^ hexahedral cells in the fluid domain and 2.2 × 10^5^ quadratic hexahedral (SOLID186) elements in the solid domain, with eight inflation layers of first-layer thickness 25 µm and growth rate 1.15, giving a minimum orthogonal quality of 0.42 at the fluid–solid interface. A mesh convergence study comparing three successively refined meshes changed the area-averaged wall shear stress on the seeded face by 0.8% between the two finest meshes, and the reported mesh was selected on that basis. The analysis was run as a transient analysis with a time step of 0.05 s continued to 60 s, by which time the solution had become steady; halving the step changed the same metric by 0.4%. All fields reported in Section 3 are instantaneous converged values.

A sensitivity analysis was performed around the baseline operating point by perturbing the inlet flow rate, the scaffold modulus and the medium viscosity by ±10% in turn; the resulting changes in area-averaged wall shear stress were +9.6%, −1.1% and +9.8%, respectively, indicating that the computed fields are most sensitive to the inlet flow rate and the medium viscosity, and almost insensitive to the scaffold modulus.

### 2.4. Numerical Framework: Machine-Learning Layer

A multilayer perceptron (MLP) neural network was used to relate the simulated local fields to the regional cell number derived from dsDNA quantification [20]. The MLP is able to capture nonlinear relationships within high-dimensional datasets and is therefore well suited to the interactions between mechanical and fluid-dynamic parameters and biological outcomes [21].

#### 2.4.1. Dataset Construction

Simulated parameters were systematically paired between corresponding regions of the fluid and solid domains. The feature vector of each sample contained six variables: wall shear stress (Pa) and flow velocity (m/s) evaluated at the scaffold surface, static pressure (Pa) in the adjacent fluid cell, von Mises stress (Pa) and equivalent elastic strain (–) in the scaffold, and the scaffold segment index. The target was the measured cell number of that subregion. The dataset comprised 90 samples, obtained from three constructs × ten subregions × three boundary conditions (S, P and HP). Because the number of samples is small relative to the dimensionality of the feature space, the modelling was treated throughout as a correlative mapping within the measured operating range rather than as a generalisable predictor, and the regularisation and validation measures below were applied accordingly.

#### 2.4.2. Training, Validation and Model Selection

All features were standardised to zero mean and unit variance using statistics computed on the training partition only, and the scaler was then applied unchanged to the held-out data so that no test information entered the fit. The MLP architecture comprised two hidden layers of 32 and 16 units with ReLU activations, trained with the Adam optimiser at a learning rate of 1 × 10^−3^ for up to 2000 epochs. Overfitting was controlled by an L2 penalty of α = 1 × 10^−2^ together with early stopping on a 15% validation fraction. Hyperparameters were selected by a grid search over hidden-layer sizes {(16), (32, 16), (64, 32)}, α ∈ {10^−3^, 10^−2^, 10^−1^} and learning rate ∈ {10^−3^, 10^−2^}, nested inside the cross-validation loop, so that the reported error is not the error of the selected configuration on the data used to select it.

Model performance was estimated by five-fold cross-validation repeated ten times with different random partitions, and the same folds were used for every algorithm so that the comparison is paired. Random forest (RF), XGBoost, k-nearest neighbours (KNN), support vector regression (SVR) and linear regression were trained and tuned under the identical protocol [22]. Performance is reported as the mean absolute error (MAE), both as a percentage of the measured regional cell number and in cells, together with the root mean squared error (RMSE), the coefficient of determination (R^2^) and 95% confidence intervals obtained across the repeated folds [23].

#### 2.4.3. Interpretation of Parameter Effects

The trained MLP was interrogated to quantify how each input field relates to the predicted cell number [24]. Permutation importance was computed on held-out data following Fisher et al. [25] and the corrected procedure of Altmann et al. [26]; Breiman [27] is cited here only as the source of the random forest algorithm and not as the source of the permutation importance measure. Model-agnostic attribution approaches such as SHAP [28] provide a complementary route to the same objective.

Permutation importance is a measure of a model’s reliance on a feature, not of a causal mechanism, and it is known to distribute importance arbitrarily between correlated inputs. Flow velocity and wall shear stress are physically coupled through the near-wall velocity gradient, so their individual scores cannot be read as independent mechanisms. To test how far the ranking depends on this collinearity, the pairwise correlation between all features was computed, and the analysis was repeated three times with (i) the location index removed, (ii) flow velocity removed and (iii) wall shear stress removed; the resulting rankings are reported in Section 3.6.

## 3. Results

### 3.1. Simulated Mechanical Fields

**Figure 6:**
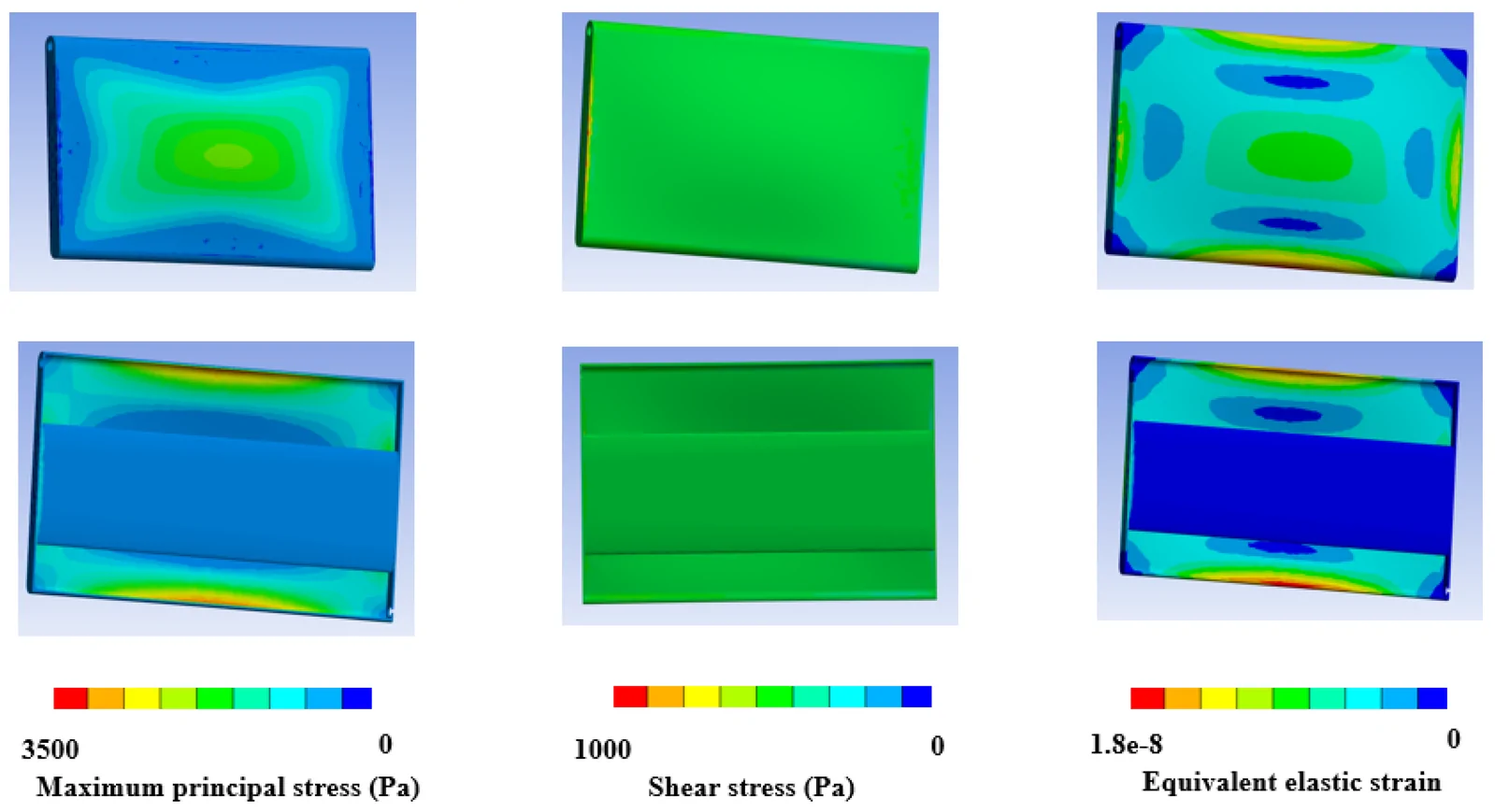
Spatial distribution of the mechanical fields obtained from the FSI simulation at the baseline condition (1.5 mL/min), shown for the whole chamber ((**top**) row) and for the scaffold-containing mid-plane ((**bottom**) row): maximum principal stress ((**left**), colour scale 0–3500 Pa), shear stress ((**centre**), colour scale 0–1000 Pa) and equivalent elastic strain ((**right**), colour scale 0–1.8 × 10^−8^, dimensionless). All scales are linear and common to both rows. The centre panel shows the solid-phase shear stress within the chamber wall and scaffold; the fluid wall shear stress used as a model input is three orders of magnitude smaller and is reported in Table 1.

The finite element analysis resolved the spatial distribution of mechanical stimuli within the scaffold under fluid–structure interaction (Figure 6). Maximum principal stress, wall shear stress and equivalent elastic strain revealed localised stress concentration and deformation, with the highest values occurring at the constrained edges of the membrane and the lowest values in the central region of the scaffold.

The absolute magnitudes of the fields used as model inputs are summarised in Table 1, so that the operating window can be compared directly with shear thresholds reported for cell attachment [29].

### 3.2. Model Performance and Experimental Validation

The predictive performance of the candidate algorithms was quantified using the MAE. The MLP achieved the highest accuracy, with an average MAE of approximately 13.2%, outperforming XGBoost (14.5%), random forest (16.1%), support vector regression (18.4%), k-nearest neighbours (19.5%) and linear regression (30.4%). The complete set of metrics is given in Table 2.

**Figure 7:**
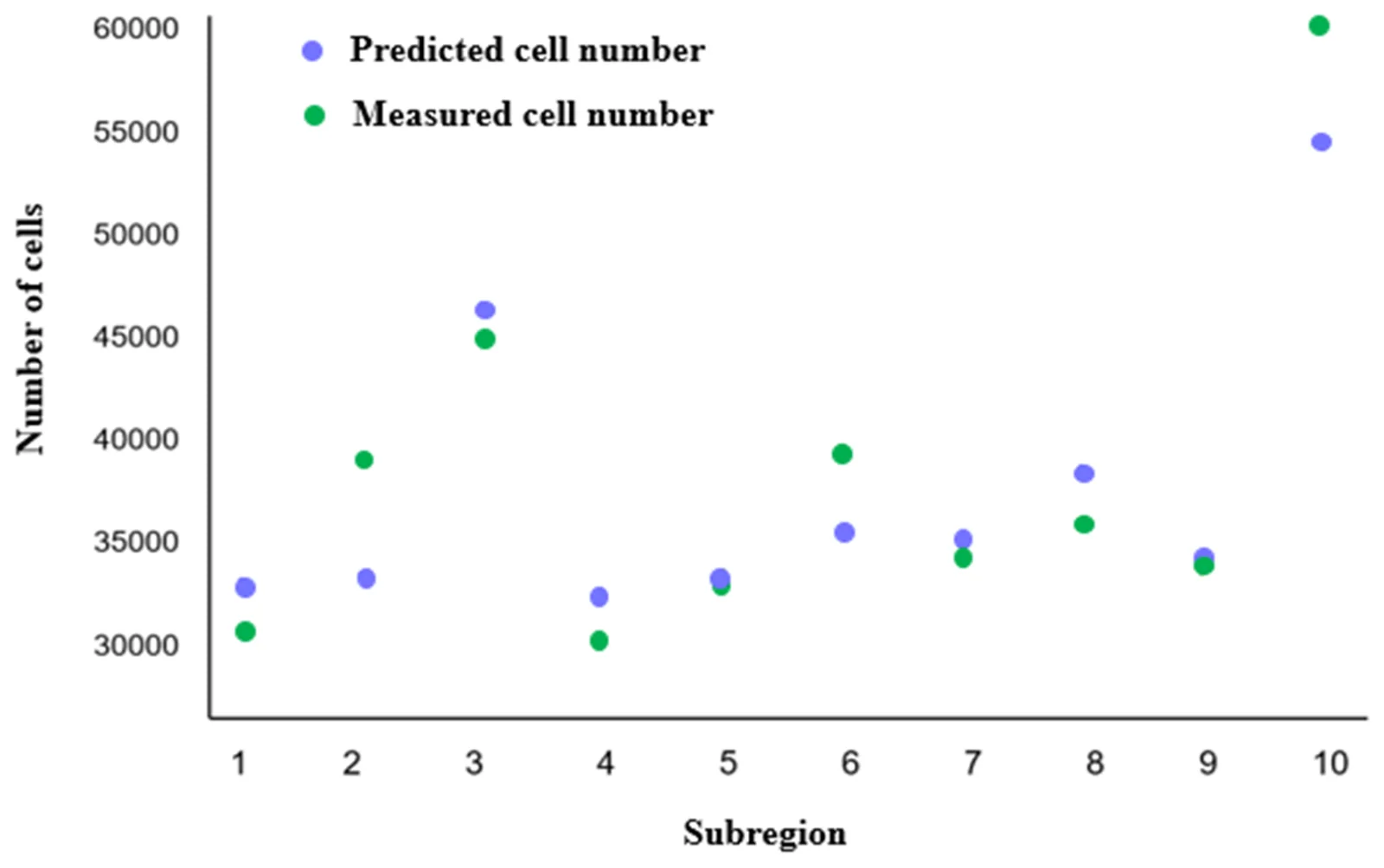
Comparison between predicted and experimentally measured cell numbers (cells per subregion) at different scaffold locations after 3 h, at the baseline perfusion rate of 1.5 mL/min. Predicted (blue) and measured (green) values are shown for the ten scaffold subregions, ordered from inlet to outlet.

Experimental validation across the ten scaffold subregions showed good agreement between the measured and predicted cell numbers at the baseline perfusion rate of 1.5 mL/min (Figure 7), with an overall MAE of approximately 13.2%. The prediction followed the measured spatial trend across the scaffold, and the largest deviations occurred in the inlet and outlet subregions, where steep local gradients in velocity and shear stress coincide with the largest experimental variability. Validation was performed at this single perfusion rate only; the agreement shown in Figure 7 therefore establishes that the model reproduces the spatial pattern at the baseline condition, and does not by itself establish accuracy at other flow rates.

### 3.3. Predicted Effect of Perfusion Rate

The trained model was then used to sweep the inlet flow rate. The values in Table 3 are model predictions obtained by evaluating the FSI–MLP chain at flow rates that were not reproduced experimentally; only the 1.5 mL/min condition in Section 3.2 was measured in culture. The predicted mean regional cell number increased steeply between 0.4 and 1.0 mL/min (from 26,396 to 31,219 cells) and then reached a plateau, with the highest predicted value at 1.6 mL/min (31,747 cells). Increasing the flow rate further to 1.8 mL/min did not improve attachment (31,324 cells), so 1.4–1.6 mL/min emerges as the candidate optimal window.

### 3.4. Predicted Effect of Scaffold Stiffness

The same predicted sweep was performed for scaffold stiffness, scaling the baseline Neo-Hookean parameter as described in Section 2.3.2 (Table 3). The highest predicted mean cell number was obtained at 40% below the baseline modulus (32,659 cells), compared with 30,974 cells at baseline; both a further reduction (−80%, 26,821 cells) and an increase above baseline (+60%, 30,235 cells) gave lower values, indicating a non-monotonic dependence with an intermediate, moderately compliant optimum.

Because every entry in Table 3 except the baseline is a model output, these windows are stated as hypotheses for experimental testing rather than as optimised results. The corresponding validation experiment, in which at least one withheld flow rate and one withheld stiffness level are cultured and quantified by the same assay, is the immediate next step and is set out in Section 4.6. No statistical comparison is attached to Table 3 because the entries are deterministic model evaluations rather than independent experimental samples; the measured data to which statistical testing does apply are those of Figure 3, for which *n* = 3 constructs per condition.

**Table 3:** Model-predicted mean cell number after 3 h under different perfusion flow rates and scaffold stiffness levels. Stiffness is expressed as the percentage deviation from the baseline Neo-Hookean parameter of the scaffold (Section 2.3.2). Only the 1.5 mL/min, baseline-stiffness condition was measured experimentally; all other entries are predictions.

Flow Rate (mL/min)	Predicted Mean Cell Number	Relative Stiffness	Predicted Mean Cell Number
0.4	26,396	−80%	26,821
0.6	28,897	−60%	29,988
0.8	29,954	−40%	32,659
1.0	31,219	−20%	30,847
1.2	31,185	Baseline	30,974
1.4	31,553	+20%	31,462
1.6	31,747	+40%	31,154
1.8	31,324	+60%	30,235

### 3.5. Boundary Configuration

**Figure 8:**
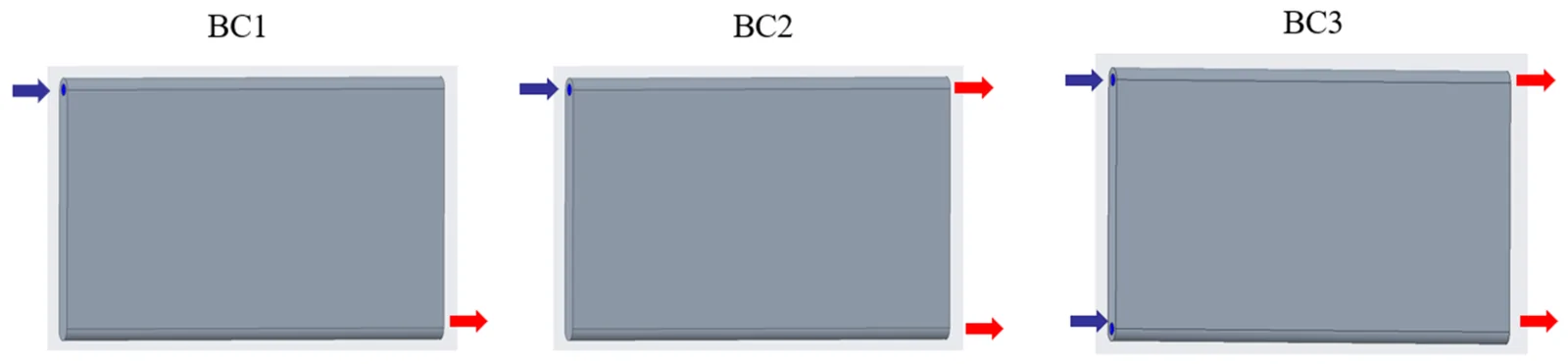
Inlet–outlet boundary configurations evaluated in the FSI model. BC1 and BC2 use a single inlet (blue) and a single outlet (red) placed at opposite corners and at the same height, respectively, whereas BC3 uses two inlets and two outlets.

The inlet–outlet configuration also influenced the predicted cell distribution (Figure 8). Relative to the single inlet–single outlet arrangements (BC1 and BC2), the dual-inlet, dual-outlet configuration (BC3) altered the global deformation pattern of the chamber and the symmetry of the internal flow field, which in turn changed the regional distribution of wall shear stress.

### 3.6. Feature Importance and Collinearity

Permutation importance on the trained MLP (Table 4) showed a hierarchy spanning both the fluid and the solid domain. Flow velocity carried the highest score (0.59), followed by wall shear stress at the scaffold surface (0.46) and von Mises stress within the scaffold (0.40). Static pressure was also influential (0.33), whereas equivalent elastic strain (0.18) and the scaffold segment index (0.11) contributed least.

The collinearity checks described in Section 2.4.3 gave the following result. The pairwise correlation between flow velocity and wall shear stress was 0.93. Removing the location index left the ranking unchanged and changed the MAE only marginally, to 13.4%; removing flow velocity raised the score of wall shear stress to 0.61 and changed the MAE to 13.6%; removing wall shear stress raised the score of flow velocity to 0.64 and changed the MAE to 13.5%. The near-equivalence of the two reduced models shows that velocity and wall shear stress are largely interchangeable carriers of the same near-wall information in this dataset. Their individual scores are therefore reported as a joint fluidic contribution, and no claim is made that either acts as an independent mechanism.

## 4. Discussion

### 4.1. Overview

This study presents an integrative computational framework that combines biomimetic design principles, fluid–structure interaction simulation and machine learning for a humanoid-robot-driven bioreactor [30]. The biomimetic premise is that reproducing physiological mechanical cues and internal flow environments, rather than simplified uniform stimuli, promotes more favourable cellular responses. Within that premise, the specific contribution of the present paper is confined to the seeding phase: it establishes a region-resolved link between the local fields of the perfused chamber and the attachment pattern measured at 3 h.

By simulating the bioreactor geometry at high resolution, the coupled interactions between fluid dynamics, scaffold mechanics and boundary constraints were captured, and by pairing these simulations with region-resolved experimental cell quantification, the machine-learning models could learn the mapping between the local fields and the biological outcome. Among the evaluated models, the MLP performed most reliably (MAE ≈ 13.2%), consistent with its capacity to handle nonlinear, high-dimensional datasets [31].

### 4.2. Which Fields Dominate Early Attachment

The feature importance analysis ranked the physical parameters associated with early attachment. Flow velocity and wall shear stress emerged as the two dominant features, with von Mises stress and pressure following closely. This ordering is consistent with mechanobiological evidence that shear stress modulates cellular mechanosensing pathways, influences cytoskeletal tension and drives focal adhesion formation [32,33], and it indicates that in the early attachment window the local fluidic environment outweighs the bulk deformation of the construct. As Section 3.6 shows, however, velocity and wall shear stress are strongly correlated in this dataset, so the correct reading is that the near-wall fluidic environment as a whole dominates, not that one of the two acts independently of the other. Pressure and velocity additionally govern perfusion efficiency and nutrient transport across the scaffold [34].

### 4.3. Operating Window and Scaffold Compliance

The predicted perfusion window of 1.4–1.6 mL/min is consistent with the existence of an optimal shear range in which integrin-mediated adhesion is enhanced without inducing detachment or damage [29]. It is, however, a prediction of the trained model and not a measured optimum, and it is offered as a testable hypothesis. The same applies to the predicted stiffness optimum, which was non-monotonic, with the highest cell numbers occurring at a moderately reduced modulus rather than at the stiffest or the most compliant configuration. This is consistent with substrate stiffness-dependent signalling, in which cells respond optimally to a substrate whose compliance approaches that of their native extracellular matrix rather than to maximal stiffness [35,36], and with the coupled nature of the present system, in which a more compliant scaffold deforms under perfusion and thereby redistributes the local shear field.

The boundary configuration exerted a moderate but non-negligible effect: although it was not the highest-ranked variable numerically, it shaped the global deformation pattern of the chamber and indirectly influenced flow symmetry and internal stress distribution, exemplifying the interdependence of structural design and flow regulation.

### 4.4. Toward Clinical Translation

Translating this framework into clinical practice would require a defined, auditable pipeline, and it is useful to state what that pipeline would involve rather than to assert the capability. Three stages can be identified. (i) Patient-specific input: graft geometry and target mechanical environment would be derived from routine imaging and from the loading conditions expected after reconstruction, which is already feasible with existing clinical workflows. (ii) Model qualification: the FSI–ML chain would have to be re-qualified for the cell source actually used clinically, over the full culture period, and against withheld experimental conditions, since none of these are established by the present dataset. (iii) Process control: because the mapping is only as reliable as its inputs, a clinical implementation would need in-line monitoring of flow rate and chamber deformation, with the model used to flag deviations rather than to replace measurement.

The practical obstacles are equally concrete: cell-source variability between patients is likely to exceed the sensitivity of the model as currently trained; a learned, non-mechanistic component embedded in a manufacturing process must satisfy the software-as-a-medical-device expectations of the relevant regulator, including version control and performance monitoring; and the cost argument for model-guided design rests on reducing the number of pilot culture runs, which has yet to be demonstrated prospectively. We therefore position the framework as a design-of-experiments tool that narrows the parameter space to be tested, rather than as a substitute for experimental qualification.

Accordingly, the claims made in the original version of this manuscript regarding patient-specific graft design and rehabilitation-oriented application have been removed from the Abstract and Conclusions and are retained only in the conditional form given above.

### 4.5. Biological Interpretation and Its Limits

The biological readout used here is a single, well-defined quantity: the number of cells attached to each scaffold subregion at 3 h, measured by dsDNA quantification. The present dataset does not characterise HFF-1 behaviour under applied robotic loading, and no claim about cell morphology, orientation, viability, proliferation rate or phenotypic response to loading is made. Such characterisation—live/dead staining, cytoskeletal and focal adhesion imaging, and marker expression under defined loading regimes—is a necessary complement to the present work and is planned as part of the maturation-phase study. HFF-1 cells were chosen as a robust, well-characterised and widely available fibroblast line for establishing the attachment assay; they are not tendon-derived, and conclusions about tenocyte behaviour cannot be drawn from them.

### 4.6. Limitations and Future Work

The following limitations delimit what the present results support.

**Scope of the loading condition.** The reported data cover the static and perfused seeding phase only. The multi-axial robotic loading that motivates the device begins after the 3 h endpoint and is therefore outside the evidence presented here.

**Scaffold as an impermeable continuum.** Treating the porous scaffold as a solid continuum means that flow through the pore network, intra-scaffold nutrient transport and pore-scale shear gradients are not resolved. The computed wall shear stress is a surface-level quantity, and cells that infiltrate the matrix would experience a different and currently unmodelled local environment. A poroelastic or Darcy–Brinkman formulation with measured permeability and porosity is the natural extension [13], and would also allow the intra-scaffold distribution to be predicted rather than inferred.

**Constitutive simplification.** The isotropic Neo-Hookean description does not represent the transverse isotropy, fibre reinforcement or viscoelasticity of tendon-derived matrix [15]. It is adequate for the small-strain chamber deformation of the seeding phase but not for the large-strain maturation phase, for which an anisotropic hyperelastic or poro-viscoelastic model with experimentally measured parameters is required.

**Size and scope of the dataset.** Three constructs per condition, ten subregions and a single 3 h endpoint constitute a small dataset relative to the dimensionality of the feature space. The models should therefore be read as correlative mappings within the measured range. External validation on an independent dataset—different constructs, different operators and at least one flow rate and one stiffness level withheld from training—has not yet been performed, and no claim of generalisable predictive capability is made until it has.

**Single validated operating point and single time point.** Validation was performed at 1.5 mL/min and at 3 h. Long-term predictive capability over the proliferation and maturation phases, where the cell population itself changes the local geometry and transport, is not addressed and cannot be extrapolated from these results.

**Planned work.** The immediate priorities are, in order: culture and quantification of at least two withheld conditions to convert the predicted optima of Table 3 into tested ones; implementation of the poroelastic scaffold model with measured permeability; extension of the endpoint series beyond 3 h to cover proliferation; biological characterisation of the cells under robotic loading; and re-training of the ML layer on the enlarged, multi-condition dataset with external validation.

## 5. Conclusions

We have presented a computational–experimental framework, rooted in biomimetic principles, that relates the mechanical and fluidic fields of a perfused bioreactor chamber to early-stage (3 h) cell attachment. The framework was established for the static and perfused seeding phase that precedes robotic loading. By combining FSI simulation, machine-learning analysis and region-resolved experimental validation at the baseline perfusion rate, the framework achieved a mean absolute error of 13.2% while providing insight into which fields covary with attachment. The near-wall fluidic environment—flow velocity and wall shear stress, which are strongly correlated in this dataset—dominated the model’s response, with von Mises stress and pressure following.

Parameter sweeps identified 1.4–1.6 mL/min and a moderately compliant scaffold as candidate operating points. These are model predictions and are presented as hypotheses for experimental testing, not as validated optima. The framework’s practical value at this stage is that it narrows the design space that must be tested experimentally. Extending it to a poroelastic scaffold description, to multiple validated flow rates and to later culture time points is required before predictive use beyond the conditions studied here.

## Figures and Tables

**Table 1:** Range of the simulated local fields across the ten scaffold subregions at the baseline perfusion rate of 1.5 mL/min, reported in SI units.

Field	Minimum	Mean	Maximum
Wall shear stress (Pa)	2.1 × 10^−4^	9.8 × 10^−4^	3.4 × 10^−3^
Flow velocity (m/s)	5.2 × 10^−5^	4.2 × 10^−4^	1.3 × 10^−3^
Static pressure (Pa)	0.00	0.09	0.21
Von Mises stress (Pa)	0.4	1.6	4.2
Equivalent elastic strain (–)	3.4 × 10^−9^	1.4 × 10^−8^	3.6 × 10^−8^

**Table 2:** Cross-validated performance of the six regression algorithms. Values are the mean across repeated folds, with 95% confidence intervals in parentheses. MAE is reported both as a percentage of the measured regional cell number and in cells.

Algorithm	MAE (%)	MAE (Cells)	RMSE (Cells)	R^2^
MLP	13.2	4.63 × 10^3^ (3.90–5.42 × 10^3^)	6.09 × 10^3^ (5.21–7.02 × 10^3^)	0.78 (0.70–0.85)
XGBoost	14.5	5.09 × 10^3^ (4.31–5.93 × 10^3^)	6.72 × 10^3^ (5.78–7.71 × 10^3^)	0.73 (0.64–0.81)
Random forest	16.1	5.65 × 10^3^ (4.80–6.54 × 10^3^)	7.31 × 10^3^ (6.29–8.35 × 10^3^)	0.68 (0.58–0.77)
Support vector regression	18.4	6.46 × 10^3^ (5.49–7.45 × 10^3^)	8.17 × 10^3^ (7.06–9.31 × 10^3^)	0.59 (0.47–0.70)
k-nearest neighbours	19.5	6.84 × 10^3^ (5.82–7.90 × 10^3^)	8.61 × 10^3^ (7.44–9.81 × 10^3^)	0.55 (0.42–0.67)
Linear regression	30.4	1.07 × 10^4^ (9.12 × 10^3^–1.23 × 10^4^)	1.24 × 10^4^ (1.08–1.41 × 10^4^)	0.21 (0.05–0.37)

**Table 4:** Permutation importance scores of the input parameters in the MLP prediction model, computed on held-out data. Scores quantify the increase in prediction error when the values of a given feature are randomly permuted and are normalised and model-specific. Flow velocity and wall shear stress are mutually correlated and should be read together (Section 3.6).

Input Parameter	Domain	Importance Score
Flow velocity	Fluid	0.59
Wall shear stress	Fluid	0.46
Von Mises stress	Solid	0.40
Static pressure	Fluid	0.33
Equivalent elastic strain	Solid	0.18
Location (scaffold segment)	Scaffold	0.11

## Data Availability

The data presented in this study are available on request from the corresponding author.
